# Refeeding Hypophosphatemia During ECMO Support: A Retrospective Cohort Study on Incidence, Predictors, and Association with Outcome

**DOI:** 10.3390/nu18213491

**Published:** 2026-10-23

**Authors:** Patrick Haselwanter, Max Oberweger, Sarah Schwaiger, Marlene Hintersteininger, Marlene Riedl-Wewalka, Mathias Maleczek, Daniel Laxar, Martina Hermann, Nina Buchtele, Thomas Staudinger, Christian Zauner, Mathias Schneeweiss-Gleixner

**Affiliations:** 1 Department of Medicine III, Division of Gastroenterology and Hepatology, Intensive Care Unit 13H1, Medical University of Vienna, Waehringer Guertel 18–20, 1090 Vienna, Austria; patrick.haselwanter@meduniwien.ac.at (P.H.);; 2 Department of Anesthesia, Intensive Care Medicine and Pain Medicine, Medical University of Vienna, 1090 Vienna, Austria; 3 Ludwig Boltzmann Institute for Digital Health and Patient Safety, Medical University of Vienna, 1090 Vienna, Austria; 4 Department of Medicine I, Intensive Care Unit 13i2, Medical University of Vienna, 1090 Vienna, Austria

**Keywords:** refeeding syndrome, hypophosphatemia, extracorporeal membrane oxygenation, ECMO, critical care, medical nutrition therapy, COVID-19

## Abstract

**Background/Objectives:** Refeeding syndrome (RFS) is a potentially life-threatening complication of nutritional support in critically ill patients. Its incidence and clinical relevance in patients requiring extracorporeal membrane oxygenation (ECMO) are unknown. We retrospectively investigated the incidence, characteristics and clinical relevance of RFS in COVID-19 patients receiving venovenous (vv-)ECMO support. **Methods:** In a secondary analysis of 101 COVID-19 patients treated with vv-ECMO at a tertiary center, RFS was defined by a decrease of more than 0.16 mmol/L in serum phosphate to a value below 0.65 mmol/L within seven days of starting medical nutrition therapy (MNT), or a pre-existing phosphate below 0.65 mmol/L. Electrolyte trajectories, nutritional delivery, predictors of RFS, and the association with ICU mortality were analyzed. Robustness was assessed across alternative published definitions. **Results:** RFS developed in 46 of 101 patients (45.5%), with an incidence ranging from 23.8% to 84.2% depending on the definition applied. Patients with RFS had a lower body mass index and required significantly more phosphate substitution than those without (93.5% vs. 43.6%; *p* < 0.001). Early energy delivery did not differ between groups and was not associated with RFS. RFS was not associated with ICU mortality (41.3% vs. 41.8%; adjusted hazard ratio 0.80, *p* = 0.51), a finding consistent across all definitions examined. **Conclusions:** In this COVID-19 vv-ECMO cohort, refeeding-associated hypophosphatemia was frequent but not associated with caloric delivery or ICU mortality under close electrolyte monitoring. These hypothesis-generating findings do not support reducing caloric intake for isolated hypophosphatemia and require prospective confirmation.

## 1. Introduction

Refeeding syndrome (RFS) is a potentially life-threatening complication in malnourished patients after the restart of nutrition. Due to metabolic alterations during starvation, rapid glucose/nutrient supply leads to the release of insulin, with intracellular shifts in phosphate, magnesium, and potassium. As a result, low serum electrolyte levels (i.e., hypophosphatemia, hypomagnesemia, and hypokalemia) are key clinical determinants of RFS, with potentially severe consequences for cardiac and respiratory function if not recognized [1]. Moreover, it is characterized by thiamine deficiency, as thiamine is a key cofactor in glucose-dependent metabolism. Therefore, restarting medical nutrition therapy (MNT) after periods of starvation should always be done with caution, close monitoring of electrolyte levels, and adequate thiamine replacement [2]. Patients predisposed to malnutrition are at particular risk of RFS, yet are often overlooked in clinical practice. These include patients with disorders of nutrient uptake (e.g., inflammatory bowel disease, short bowel syndrome) and metabolism (e.g., cirrhosis), as well as patients admitted to the intensive care unit (ICU) [3,4,5]. The incidence of RFS in the ICU has been reported to range from 14.6% to 52%, depending on the cohort and the RFS definition [6,7,8,9,10,11].

A standardized definition of RFS is currently lacking. While the American Society for Parenteral and Enteral Nutrition (ASPEN) definition includes all three electrolyte depletions within 5 days after MNT start [1], Friedli et al. and Rio et al. also include clinical symptoms, such as edema, tachycardia, tachypnoea, and organ dysfunction [12,13]. In critically ill patients, these clinical signs are often influenced by the critical illness itself and ICU-specific treatment strategies (e.g., fluid management and extracorporeal therapies) that also affect electrolyte balance. This heterogeneity in cohorts and definitions makes systematic extraction of patients with RFS difficult. The definition by Marik et al. primarily focuses on hypophosphatemia (i.e., serum phosphate levels < 0.65 mmol/L within 7 days after MNT) and may be the most feasible for ICU patients [6]. Most ICU cohorts investigating RFS comprise heterogeneous populations of critically ill patients. Although a recent consensus statement by the Extracorporeal Life Support Organization (ELSO) recommends monitoring for RFS during ECMO, it provides no guidance on its incidence, definition or clinical relevance in this population, reflecting the absence of data [14]. This is further complicated in the ECMO setting, where renal replacement therapy (RRT) is frequently required and independently lowers serum phosphate, potentially confounding the assessment of RFS [15]. While the impact of undernutrition in patients with coronavirus disease 2019 (COVID-19) receiving venovenous (vv-)ECMO has been described previously [16], the incidence and clinical relevance of RFS in this population remain unknown. We therefore investigated RFS during vv-ECMO therapy at a large tertiary center.

## 2. Materials and Methods

### 2.1. Study Design and Cohort

This is a secondary analysis of a previously described cohort of adult patients requiring venovenous extracorporeal membrane oxygenation (vv-ECMO) support for COVID-19-associated acute respiratory distress syndrome at the Medical University of Vienna, a large tertiary care center in Austria [16,17]. All patients treated between March 2020 and May 2021 were included. Patients who did not receive medical nutrition therapy (MNT) during ECMO support were excluded. The observation period was set from the first day of MNT to ICU discharge or death. The study is reported in accordance with the STROBE statement for observational studies.

This study was approved by the local Ethics Committee of the Medical University of Vienna (ethics vote number: 2440/2020) and performed in accordance with the Declaration of Helsinki. Given the study’s observational design, the requirement of informed consent was waived by the local Ethics Committee.

### 2.2. Nutrition-Related Data and Refeeding Syndrome

All patients received nutrition support according to the ICU’s standard operating procedures and the European Society for Clinical Nutrition and Metabolism (ESPEN) guidelines, aiming to start MNT as soon as possible via nasogastric tube after ECMO cannulation. Enteral nutrition (EN) was the preferred route of administration. Parenteral nutrition (PN) was used only in patients with contraindications to EN or insufficient EN. The treating physicians set nutrition goals, estimating daily energy requirements using a simple weight-based equation (25 kcal per kg body weight per day, using adjusted body weight for a body mass index (BMI) of ≥30 kg/m^2^) [4].

RFS was defined as a decrease of more than 0.16 mmol/L in serum phosphate concentrations to values below 0.65 mmol/L within seven days after the start of MNT, or pre-existing serum phosphate levels below 0.65 mmol/L [11]. This represents a purely biochemical definition. Clinical signs of RFS (i.e., edema, arrhythmia, respiratory and organ dysfunction), as typically used in other RFS definitions, were not included due to strong confounding factors in critically ill ARDS patients during vv-ECMO [12,13]. Serum phosphate, potassium, and magnesium were assessed once daily. To assess the robustness of our findings, the cohort was additionally classified according to alternative published definitions, highlighted in Supplementary Table S1 [1,6,18]. To investigate the rate of RFS and differences in the clinical management of RFS patients compared to patients without RFS, we documented electrolyte levels, electrolyte and vitamin substitutions, MNT, and energy delivery daily for seven days after the commencement of MNT. To illustrate trajectories of serum electrolyte levels, electrolyte substitution, energy and protein delivery, we also plotted daily values over the first 14 days of MNT. Electrolytes were monitored daily and substituted by the treating physician. Phosphate was replaced intravenously with 20 mmol phosphate for serum phosphate <0.65 mmol/L and with 40 mmol phosphate for severe hypophosphatemia, but with no fixed protocol.

### 2.3. Data Collection

The Vienna General Hospital uses a digital patient data management system (IntelliSpace Critical Care and Anesthesia, Philips, Amsterdam, The Netherlands), which enables complete documentation of all crucial patient characteristics, including diagnosis, physical examination, vital signs, laboratory tests, ICU-specific therapy, and medication. The severity of critical illness was calculated using the Sequential Organ Failure Assessment Score (SOFA) and the Simplified Acute Physiology Score II (SAPS II) [19,20]. Data on laboratory results (especially electrolytes), life-sustaining therapies (i.e., mechanical ventilation, vasopressor support, and RRT), electrolyte and vitamin substitution, and MNT were documented over seven days after the commencement of nutrition support.

### 2.4. Statistical Analysis

The primary aim of this study was to assess the incidence of RFS in COVID-19 patients requiring vv-ECMO support. Predefined secondary objectives were to identify factors associated with the development of RFS and to evaluate its association with ICU mortality.

Qualitative parameters are shown as absolute numbers with relative frequencies (%). Quantitative parameters are presented as median with interquartile range (IQR). Categorical variables were compared using Fisher’s exact test or the chi-square test, and continuous variables using the Mann–Whitney U test. The incidence of RFS was reported with 95% Wilson confidence intervals. Factors associated with the development of RFS were assessed by univariable and multivariable binary logistic regression. The multivariable model included BMI, age, SAPS II at ECMO start, and early energy delivery (mean percentage of requirements over MNT days 1–3). In a sensitivity analysis, early energy delivery was additionally analyzed as a binary variable, dichotomized at the ESPEN adequacy threshold (≥70% vs. <70% of requirements). RRT was not entered as a covariate because only one of eleven patients receiving RRT developed RFS. It was therefore reported descriptively. The probability of ICU survival was calculated using the Kaplan-Meier product-limit method, and differences were determined by the log-rank test. The association between RFS and ICU mortality was further evaluated by Cox proportional-hazards regression (time origin: ECMO start; event: ICU death), complemented by a landmark analysis restricted to patients surviving beyond day 7 and a Fine-Gray subdistribution model accounting for the competing risk of discharge alive. A two-sided *p*-value below 0.05 was considered statistically significant. No data were missing for the analysed variables. The proportional-hazards assumption was checked with Schoenfeld residuals and held for RFS (*p* = 0.21). To assess the robustness of our findings, the incidence of RFS and its associations with ICU mortality and early energy delivery were recalculated under alternative published definitions and observation windows (Supplementary Table S1). Mortality associations were estimated using Cox models adjusted for age and BMI, and differences in early energy delivery between patients with and without RFS were compared using the Mann–Whitney U test. All statistical analyses were performed using R (Version 4.6.1, R Foundation for Statistical Computing, Vienna, Austria), IBM SPSS Statistics (Version 29.0.2.0, IBM Corp., Armonk, NY, USA), and GraphPad Prism (Version 11.0.0, GraphPad Software, San Diego, CA, USA).

## 3. Results

### 3.1. Study Population and RFS Classification

A total of 101 COVID-19 patients requiring vv-ECMO support were included. Detailed baseline and disease-severity characteristics of the total vv-ECMO cohort have already been reported by our group [16]. Briefly, the median age was 57 years (IQR: 50–62) and 72 patients (71.3%) were male. Median SOFA and SAPS II at ECMO start were 8 (IQR: 7–9) and 40 (IQR: 34–46). The median ECMO runtime was 20 (IQR: 11–31) days, with a median ICU length of stay (LOS) of 35 (IQR: 22–57) days. ICU mortality was 41.6%.

RFS developed in 46 of 101 patients (45.5%, 95% CI 36.2–55.2%). Of those, 29 (63%) met the criteria for a de novo phosphate fall from a normal baseline, while 17 (37%) had pre-existing hypophosphatemia (serum phosphate below 0.65 mmol/L on the first day of MNT).

Patients who developed RFS had a lower BMI (median 28 vs. 31 kg/m^2^, *p* = 0.032) and less frequently received RRT within the first seven days (2.2% vs. 18.2%, *p* = 0.011). ECMO runtime and ICU-LOS showed a trend towards longer values in the RFS group without reaching statistical significance (21 vs. 15 days, *p* = 0.102; and 36 vs. 34 days, *p* = 0.127). Age, sex, and ICU-specific scores did not differ between the two groups, as depicted in Table 1.

Depending on the applied definition, the incidence of RFS ranged from 23.8% to 84.2%, as highlighted in Supplementary Table S1. When RFS was restricted to a qualifying phosphate fall (strict Marik definition, fall only) or to new-onset (de novo) hypophosphatemia arising from a normal baseline, the incidence was 36.6% (37 patients) and 28.7% (29 patients), respectively (Supplementary Table S1).

### 3.2. Phosphate and Electrolyte Trajectories

**Figure 1:**
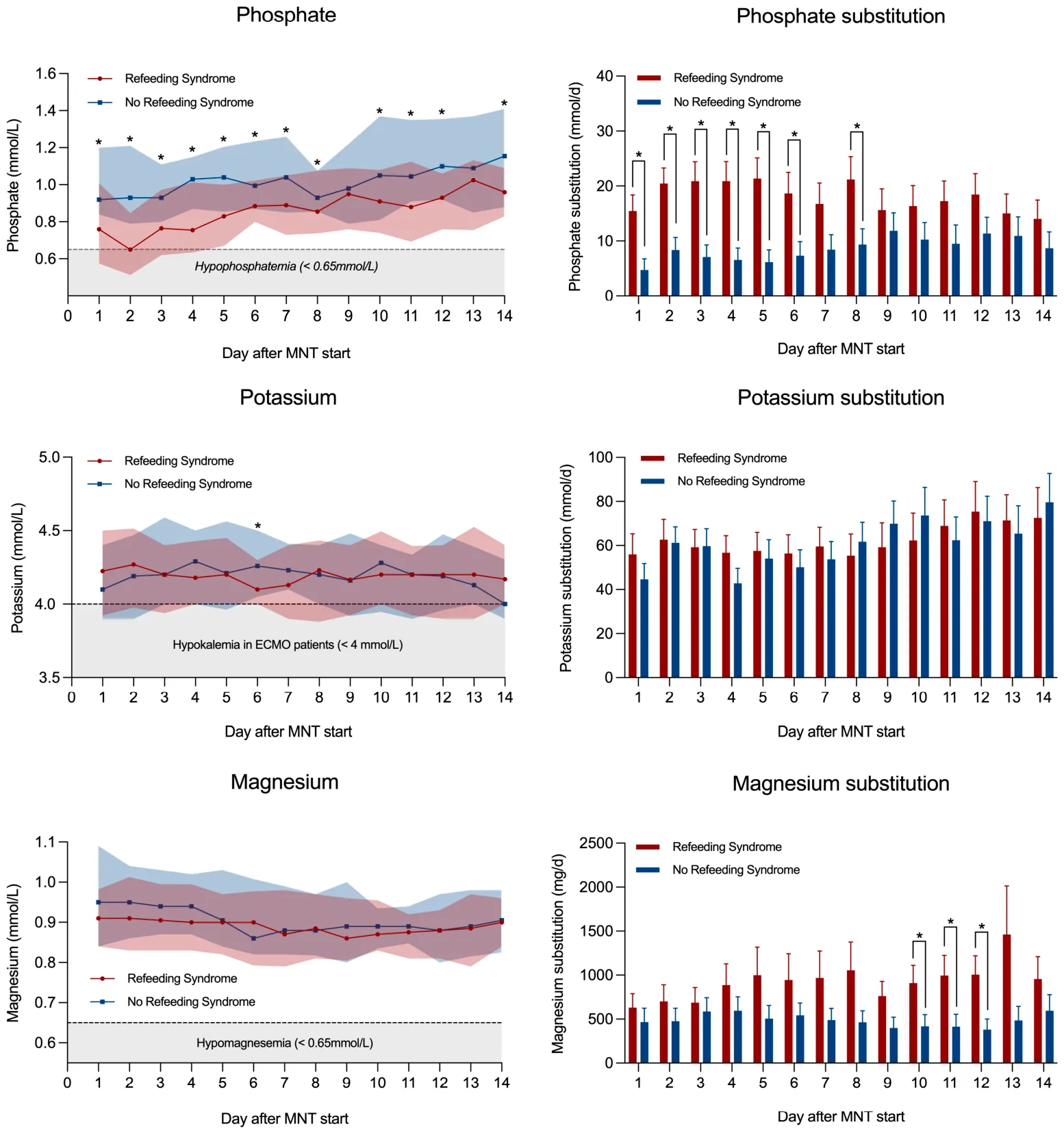
Electrolyte levels and substitution during the first 14 days of MNT in patients with or without refeeding syndrome. Serum phosphate, potassium, and magnesium concentrations during the first 14 days of medical nutrition therapy (MNT) are shown in the upper panels as medians (lines) with interquartile ranges (IQRs marked as shaded bands) for patients receiving vv-ECMO with refeeding syndrome (red) and without refeeding syndrome (blue). Daily electrolyte supplementation during the first 14 days of MNT is shown in the lower panels as means (bars) with standard errors of the mean (SEM). Statistically significant differences (*p* < 0.05) between the two groups are indicated by an asterisk (*).

Over the first 14 days of MNT, serum phosphate was lower in the RFS group than in patients without RFS, with the lowest values during the first week as depicted in Figure 1. Serum potassium and magnesium did not differ between the two groups. The electrolyte substitution showed a similar pattern. Phosphate was administered to 93.5% of RFS patients compared with 43.6% of patients without RFS (cumulative dose 80 [IQR: 50–158] vs. 0 [IQR: 0–60] mmol, both *p* < 0.001). In contrast, potassium and magnesium substitution did not differ between the two groups, as given in Table 2. Phosphate substitution in the RFS group started early (median MNT day 2) and, in de novo RFS, frequently before the phosphate nadir (median day 3). Serum phosphate recovered to ≥0.65 mmol/L by the end of the first week in 83% of these patients. Because substitution was initiated concurrently with the declining phosphate, we cannot distinguish whether substitution merely responded to the falling phosphate or also influenced its subsequent course.

### 3.3. Nutrition Support

**Figure 2:**
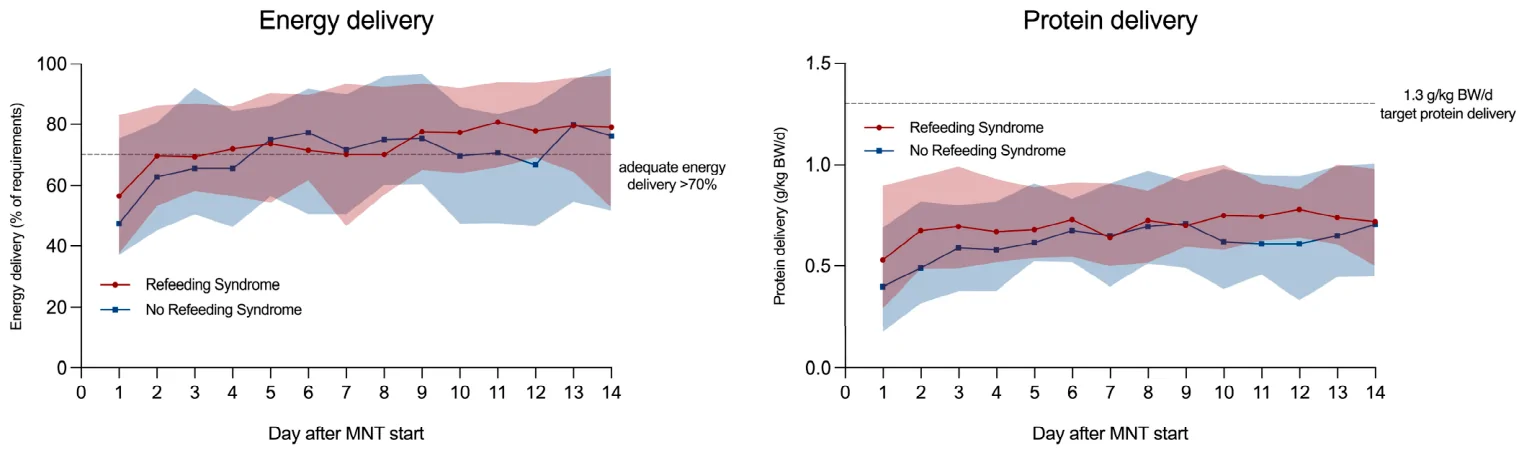
Energy and protein delivery over the first 14 days of MNT in patients with or without refeeding syndrome. Energy and protein delivery during the first 14 days of medical nutrition therapy (MNT) are shown as medians (lines) with interquartile ranges (IQRs marked as shaded bands) for patients receiving vv-ECMO with refeeding syndrome (red) and without refeeding syndrome (blue). Energy delivery is expressed as a percentage of estimated energy requirements, with the dashed line indicating adequate energy delivery (>70% of requirements). Protein delivery is expressed as g/kg body weight/day (g/kg BW/d), with the dashed line indicating the target protein delivery of 1.3 g/kg BW/d.

Overall energy and protein delivery during ECMO support in this cohort has been described previously [16,17]. Early nutritional delivery did not differ between patients with and without RFS. Median energy delivery during nutrition support days 1–3 was 64.4% in the RFS group vs. 59.2% of requirements in patients without RFS (*p* = 0.352). During nutrition support days 1–7, median energy delivery was 66.1% in the RFS group vs. 62.6% (*p* = 0.588). The proportion of days with adequate (70–100%) and inadequate (<70%) energy delivery also did not differ, as reported in Table 2 and Figure 2. Protein delivery showed a trend towards higher values in the RFS group (0.7 vs. 0.5 g/kg bodyweight/d, *p* = 0.095). Thiamine substitution did not differ between patients with and without RFS (6.5% vs. 9.1%; *p* = 0.725; Table 2).

### 3.4. Predictors of RFS Development

A lower BMI was associated with the development of RFS (OR: 0.928 [0.867–0.994] per kg/m^2^, *p* = 0.033), with a trend towards higher risk for RFS in male and older patients as depicted in the univariable logistic regression of Table 3. In the multivariable model adjusting for age, SAPS II at ECMO start, and early energy delivery, the inverse association with BMI persisted but did not reach statistical significance (adjusted OR: 0.939 [0.875–1.008] per kg/m^2^, *p* = 0.082). Early energy delivery was not associated with RFS either in the univariable or the multivariable analysis (adjusted OR: 1.002 [0.983–1.021] per % of requirements, *p* = 0.869). The results were unchanged when early energy delivery was analyzed as a binary variable regarding the ESPEN adequacy threshold (≥70% vs. <70% energy requirements; adjusted OR: 0.95 [0.40–2.2], *p* = 0.92) [4]. The independence of RFS from early energy delivery persisted under both stricter definitions (early energy delivery *p* = 0.73 for strict Marik and *p* = 0.26 for de novo RFS).

### 3.5. RFS and Clinical Outcome

**Figure 3:**
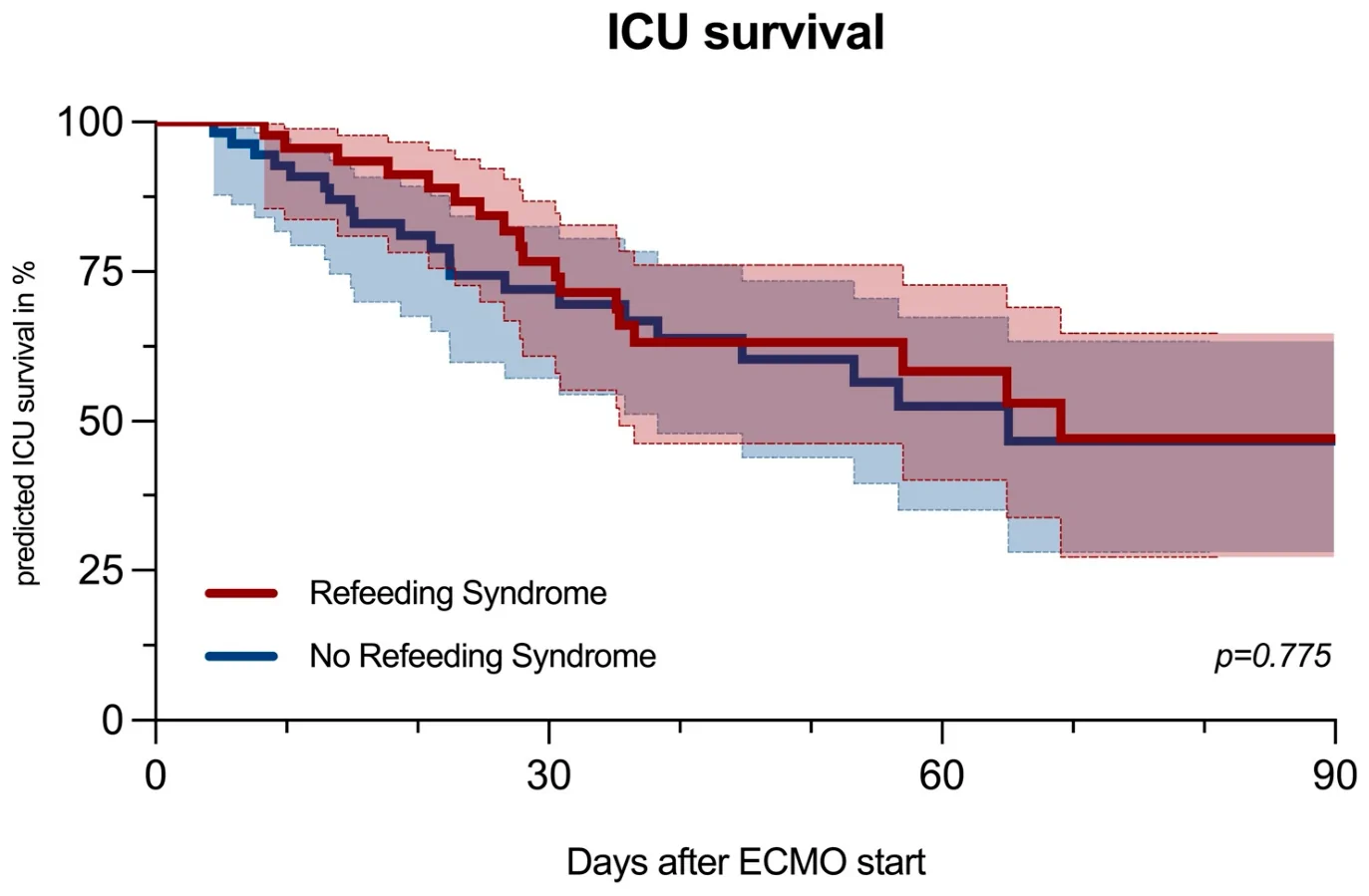
Kaplan-Meier estimates of ICU survival by RFS status. ICU survival according to refeeding syndrome status was estimated using the Kaplan-Meier method from ECMO initiation until 90 days of follow-up. Survival probabilities are shown for patients with refeeding syndrome (red) and those without refeeding syndrome (blue). The shaded areas represent the 95% confidence intervals. Survival distributions were compared using the log-rank test (*p* = 0.775).

RFS was not associated with ICU mortality. Mortality was 41.3% in patients with RFS versus 41.8% in those with no RFS (log-rank *p* = 0.775) as depicted in Figure 3. Moreover, RFS did not predict ICU mortality after adjustment for age, BMI and disease severity (adjusted HR 0.803 [0.417–1.547], *p* = 0.512) as given in the multivariable Cox regression of Table 4. This finding was confirmed in sensitivity analyses restricted to patients surviving beyond the first week and accounting for the competing risk of discharge alive (subdistribution HR: 0.91 [0.49–1.71], *p* = 0.77). In the same model, older age and higher BMI were independently associated with ICU mortality (adjusted HR 1.09 [1.03–1.16] per year and 1.07 [1.02–1.13] per kg/m2, respectively; Table 4).

The absence of an association between RFS and ICU mortality was consistent across all definitions examined, including the strict Marik (adjusted HR 1.12, 95% CI 0.58–2.14) and de novo (adjusted HR 1.01, 95% CI 0.51–2.00) definitions (Supplementary Table S1).

## 4. Discussion

In this secondary analysis of 101 COVID-19 patients requiring vv-ECMO support, RFS was frequent, occurring in almost half of the cohort. Its development was not related to the amount of caloric delivery and was not associated with ICU mortality. The only patient factor showing a trend towards an association with RFS was a lower BMI, which was not confirmed as an independent factor in the multivariable analysis. To our knowledge, this is the first study to characterize the incidence and clinical relevance of RFS in patients undergoing ECMO support. Although ECMO patients are among the most severely ill in the ICU and are a high-risk population for developing RFS, data on RFS in this population are scarce.

The incidence of RFS in critically ill patients varies considerably between studies, with reported rates ranging from 14.6% to 52% [6,7,8,9,10,11]. This variability largely reflects differences in patient populations and diagnostic criteria. Cohorts often include mixed medical-surgical or highly selected ICU subgroups, and definitions range from isolated hypophosphatemia to composite criteria requiring electrolyte disturbances together with clinical signs [6,7,8,21]. In a previous cohort of medical ICU patients from our center, applying the same phosphate-based definition, we observed a comparable incidence of 47.2% [11]. The similar rate in the present, highly selected ECMO population suggests that refeeding-associated hypophosphatemia may be common across different critically ill populations. Several factors may influence the susceptibility of ECMO patients for RFS: they are profoundly catabolic, frequently intolerant of enteral nutrition, and, in the case of COVID-19, exposed to an additional inflammatory and hypermetabolic burden that predisposes to malnutrition [16,22]. A strength of our study is its comparatively homogeneous cohort, comprising exclusively patients with COVID-19-associated ARDS on vv-ECMO, which reduces confounding by underlying diagnosis. Whether our findings can also be applied to non-COVID ECMO patients cannot be determined from this cohort. Although COVID-19 may influence the risk of RFS through its pronounced inflammatory and hypermetabolic state, the underlying refeeding mechanism is not disease-specific, and confirmation in non-COVID ECMO populations is warranted [16,22].

The lack of a uniform definition remains a central problem in the diagnosis of RFS, a fact particularly evident in the ICU setting [11]. Definitions that require clinical features such as tachycardia, tachypnoea, peripheral oedema or organ dysfunction, as proposed by Rio et al. and Friedli et al., are difficult to apply in critically ill patients, in whom these signs are ubiquitous and rarely attributable to refeeding alone [12,13]. Accordingly, the criteria of Rio et al. and Friedli et al. could not be applied in our cohort. Definitions based on electrolyte shifts are therefore more practicable, but they are not without limitations: serum phosphate is influenced by numerous factors unrelated to refeeding, including RRT, insulin and glucose administration, and acid-base disturbances [1,6,11].

In our cohort, the definition used had a significant impact on the observed RFS incidence, which ranged from 23.8% to 84.2% depending on the criteria applied, underlining how strongly reported rates depend on methodology. However, the absence of an association between RFS and ICU mortality was consistent across all definitions examined, indicating that this central finding does not depend on the definition applied. The phosphate-based criterion proposed by Marik et al. reduces this complexity to a single, objective and readily available laboratory parameter, which makes it particularly suitable for the ICU, although at the cost of not capturing the full pathophysiological spectrum of the syndrome [6].

Despite these limitations, our data suggest that the phosphate-based definition identifies a clinically meaningful patient group. Patients classified as having RFS required markedly more electrolyte substitution, indicating that the definition identified patients with an increased electrolyte demand rather than incidental laboratory alterations. Therefore, even a definition that does not encompass the full pathophysiological spectrum of RFS serves as a useful marker in the ICU to identify patients warranting close metabolic monitoring and earlier electrolyte replacement.

In this study, the development of RFS was independent of the intensity of caloric delivery. RFS is classically understood as a consequence of nutrient reintroduction, in which a carbohydrate-driven insulin secretion leads to an intracellular shift of phosphate, potassium and magnesium [1]. Against this background, one would expect higher or more rapidly advanced caloric intake to increase the risk of refeeding hypophosphatemia. However, in our cohort neither early energy delivery nor the proportion of days with adequate or inadequate intake differed between patients with and without RFS. In addition, early energy delivery was not associated with RFS in adjusted analyses. Notably, we observed the same dissociation in our previous medical ICU cohort, in which refeeding hypophosphatemia was likewise unrelated to caloric intake [11]. The replication of this finding in a second, very different critically ill population suggests that refeeding-associated hypophosphatemia in the ICU may be driven more by the patient’s underlying metabolic and nutritional state—consistent with the higher risk observed in patients with a lower BMI—than by the amount of nutrition delivered during ECMO support. RFS was independent of early energy delivery under all definitions except that of Doig et al., where patients with RFS had received more early energy [18]. This exception was limited to a definition requiring a phosphate fall and did not affect the dissociation observed under the primary and other definitions. Notably, both the decoupling from caloric delivery and the absence of a mortality association persisted across a gradient of increasingly strict refeeding definitions, from the primary definition through a qualifying-fall-only (strict Marik) to a purely de novo definition, indicating that neither finding is an artefact of including patients with pre-existing hypophosphatemia.

Many patients were referred from other hospitals specifically for ECMO therapy, and some had therefore been treated in an ICU for a longer period before our observation period began, with prior administration of supportive therapy including nutrition supply. We only collected and analyzed data concerning MNT from the initiation of ECMO onwards. Nutrition provided during the preceding ICU stay could not be investigated. Consequently, patients classified as having pre-existing hypophosphatemia may in part reflect refeeding already initiated before ECMO, and the energy delivery measured from ECMO start may not fully capture the exposure that triggered the electrolyte shift, both of which may affect the classification of RFS and its observed association with energy delivery. We regard the commencement of ECMO therapy as a clinically meaningful reference point, similar to ICU admission in studies of RFS in critically ill patients.

The second key finding of our study is that RFS was not associated with ICU mortality. This result was robust, remaining consistent after adjustment for age, BMI and disease severity and across all refeeding definitions applied. Data on the prognostic relevance of RFS in the critically ill are conflicting: while some studies have reported increased mortality [8,9,12], others, including our own previous ICU cohort, found no such association [7,10,11]. Several factors may explain this finding. RFS in our cohort was a mainly biochemical phenomenon that was timely recognized and treated. Electrolytes were monitored daily and substituted. This approach may have prevented the progression from laboratory hypophosphatemia to the clinical signs that define the syndrome in its full extent. Therefore, the absence of an association with mortality should not be interpreted as evidence that refeeding hypophosphatemia is harmless, but rather as a possible reflection of effective monitoring and treatment in this specific patient population. In addition, a higher BMI was independently associated with higher ICU mortality. This finding is in contrast with the obesity paradox reported in some COVID-19 and ECMO cohorts [23], but is in line with more recent data questioning this paradox in vv-ECMO [24]. Given the limited number of events and the observational design, this secondary finding should be regarded as exploratory. In our cohort, the median BMI was 29 kg/m^2^ (IQR: 26–35) and 47.5% of patients were obese, a distribution similar to that reported in COVID-19 ECMO populations [16,23]. The association between higher BMI and mortality persisted after adjustment for disease severity (SAPS II) and is therefore unlikely to be explained by differences in illness severity. The divergence from cohorts reporting an obesity paradox may partly reflect differences in outcome definition (in-hospital ICU mortality versus longer-term survival) and case mix.

Our study has several limitations: First, it is a retrospective, single-center analysis of a relatively small and highly selected cohort, which limits the generalizability of our findings and the statistical power for subgroup analyses. This is most evident for RRT. Although patients receiving RRT were markedly less likely to develop RFS, only one of eleven such patients met the RFS criteria, precluding meaningful adjusted analysis. This association should therefore be regarded as hypothesis-generating and cannot be resolved in the present cohort. Second, consistent with our initial ECMO cohort [16], our observation period began at ECMO initiation, and nutritional support provided during the preceding ICU stay was not available for analysis. Third, RFS was defined on the basis of serum phosphate alone. While this is practicable and objective in the ICU, it does not capture the full clinical spectrum of the syndrome, and the incidence varied substantially with the definition applied. Fourth, energy delivery remained below target in both groups (median approximately 62–66% of requirements). This limited caloric intake reduces the power to detect a dose-response relationship between energy delivery and RFS, and the absence of an association should not be interpreted as proof of independence. Fifth, because RFS classification required survival into the first week, immortal-time bias cannot be fully excluded, although a day-7 landmark analysis yielded consistent results. Sixth, serum phosphate was measured only once daily, without a fixed sampling time relative to the start of MNT. Seventh, thiamine was administered in only 7.9% of patients despite being recommended in patients at risk of RFS, and its administration did not differ between groups, reflecting local practice in which thiamine is given at the discretion of the treating team rather than prophylactically to all patients at risk. Because thiamine was administered equally in both groups, it is unlikely to have confounded the observed associations with energy delivery or mortality. However, the low overall exposure precludes any conclusion about a potential protective or modifying role of thiamine in RFS in this cohort. Finally, electrolyte substitution was not conducted by a single uniform written protocol across the participating medical and anaesthesiological ICUs, and because phosphate was replaced concurrently with the declining levels, the unsubstituted course of serum phosphate—and thus the true depth and incidence of refeeding hypophosphatemia—could not be determined. These considerations underline the need for a systematic comparison of refeeding definitions across larger and more general critically ill patient cohorts, in order to establish which criteria identify patients at risk for the development of RFS.

## 5. Conclusions

In this cohort of COVID-19 patients receiving vv-ECMO, refeeding-associated hypophosphatemia was common but, under close electrolyte monitoring and timely substitution, was not associated with caloric delivery or ICU mortality. These observations suggest that caloric intake should not be reduced solely because of isolated hypophosphatemia: ECMO patients are already at risk of underfeeding, and withholding nutrition in response to an isolated drop in serum phosphate may aggravate this deficit without benefit. This is in line with current ESPEN and ASPEN recommendations, which favour electrolyte monitoring and repletion over caloric restriction [1,4]. These conclusions must, however, be interpreted with caution: the study is retrospective and observational, RFS was defined biochemically in a single COVID-19 ECMO cohort, and—because energy delivery remained below target in both groups—the absence of an association does not prove that caloric intake and refeeding hypophosphatemia are independent. Our findings should therefore be regarded as hypothesis-generating, and prospective studies are needed to define the optimal nutritional strategy for RFS during ECMO support.

## Figures and Tables

**Table 1:** Baseline characteristics among total population and subgroups.

Baseline Characteristics	Total Population (*n* = 101)	RFS (*n* = 46)	No RFS (*n* = 55)	*p*-Value
Age in years, median (IQR)	57 (50–62)	60 (54–64)	56 (50–62)	0.051
Male, *n* (%)	72 (71.3)	37 (80.4)	35 (63.6)	0.102
BMI in kg/m^2^, median (IQR)	29 (26–35)	28 (26–31)	31 (28–36)	**0.032**
SAPS II at ECMO start, median (IQR)	40 (34–46)	40 (32–46)	40 (36–46)	0.670
SOFA at ECMO start, median (IQR)	8 (7–9)	8 (7–9)	8 (7–11)	0.719
ECMO duration in days, median (IQR)	20 (11–31)	21 (14–32)	15 (9–29)	0.102
ICU LOS in days, median (IQR)	35 (22–57)	36 (26–62)	34 (18–57)	0.127
RRT within 7 days of MNT, *n* (%)	11 (10.9)	1 (2.2)	10 (18.2)	**0.011**
ICU mortality, *n* (%)	42 (41.6)	19 (41.3)	23 (41.8)	1.000

Bold *p*-values indicate statistical significance (*p* < 0.05). Abbreviations: RFS, refeeding syndrome; BMI, body mass index; SOFA, Sequential Organ Failure Assessment, SAPS II, Simplified Acute Physiology Score II; ECMO, extracorporeal membrane oxygenation; ICU, intensive care unit; LOS, length of stay; RRT, renal replacement therapy; MNT, medical nutrition therapy; IQR, interquartile range.

**Table 2:** Nutrition support and electrolyte substitution during the first 7 days of MNT, by RFS status.

Nutrition Parameters	Total Population (*n* = 101)	RFS (*n* = 46)	No RFS (*n* = 55)	*p*-Value
**Energy & protein delivery (MNT days 1–7)**				
Energy delivery, overall (% req.) d1–3, median (IQR)	60.8 (47.5–83.0)	64.4 (54.9–84.5)	59.2 (46.1–80.3)	0.352
Energy delivery, overall (% req.) d1–7, median (IQR)	64.2 (54.3–81.3)	66.1 (55.1–81.7)	62.6 (54.0–80.7)	0.588
from enteral nutrition (% req.), median (IQR)	30.2 (12.8–49.9)	35.4 (12.2–52.1)	28.4 (15.1–45.6)	0.645
from parenteral nutrition (% req.), median (IQR)	18.1 (0.0–39.3)	19.2 (0.0–41.0)	17.7 (0.0–35.6)	0.953
Protein delivery (g/kg BW/d) d1–7, median (IQR)	0.6 (0.5–0.8)	0.7 (0.5–0.8)	0.5 (0.4–0.8)	0.095
Days with adequate energy 70–100% d1–7, median (IQR)	29 (14–57)	29 (14–57)	29 (14–57)	0.301
Days with undernutrition <70% d1–7, median (IQR)	57 (14–86)	50 (14–86)	57 (29–86)	0.445
No nutrition on ECMO day 1, *n* (%)	8 (7.9)	3 (6.5)	5 (9.1)	0.725
**Electrolyte/vitamin substitution (MNT days 1–7)**				
Phosphate substitution, any, *n* (%)	67 (66.3)	43 (93.5)	24 (43.6)	**<0.001**
Phosphate substituted (mmol), cumulative	60 (0–100)	80 (50–158)	0 (0–60)	**<0.001**
Potassium substitution, any, *n* (%)	93 (92.1)	45 (97.8)	48 (87.3)	0.068
Magnesium substitution, any, *n* (%)	49 (48.5)	26 (56.5)	23 (41.8)	0.165
Thiamine substitution, any, *n* (%)	8 (7.9)	3 (6.5)	5 (9.1)	0.725

Bold *p*-values indicate statistical significance (*p* < 0.05). Abbreviations: RFS, refeeding syndrome; MNT, medical nutrition therapy; BW, body weight; ECMO, extracorporeal membrane oxygenation; IQR, interquartile range.

**Table 3:** Univariable and multivariable binary logistic regression for predictors of refeeding syndrome.

		Univariable Analysis			Multivariable Analysis		
Variable	*n*	OR	CI of 95%	*p*-Value	Adjusted OR	CI of 95%	*p*-Value
Age (per year)	101	1.035	0.995–1.077	0.084	1.046	0.994–1.100	0.085
Male sex	101	2.349	0.943–5.850	0.067	-	-	-
BMI (per kg/m^2^)	101	0.928	0.867–0.994	**0.033**	0.939	0.875–1.008	0.082
SAPS II at ECMO start	101	0.991	0.952–1.032	0.677	0.969	0.919–1.022	0.249
SOFA at ECMO start	101	0.917	0.776–1.083	0.307	-	-	-
Energy delivery MNT days 1–3 (per % req.)	101	1.006	0.988–1.024	0.510	1.002	0.983–1.021	0.869

Bold values indicate a statistical significance (*p* < 0.05). After dichotomising early energy delivery at the ESPEN adequacy threshold results were unchanged (adjusted OR 0.95 [0.40–2.2], *p* = 0.92). Renal replacement therapy was not entered as a covariate because only 1 of 11 RRT patients developed RFS. This association is shown descriptively in Table 1. Abbreviations: OR, odds ratio; CI, confidence interval; BMI, body mass index; SAPS II, Simplified Acute Physiology Score II; MNT, medical nutrition therapy, ESPEN, European Society for Clinical Nutrition and Metabolism.

**Table 4:** Cox proportional-hazards regression for ICU mortality.

	Univariable Analysis			Multivariable Analysis		
Variable	HR	CI of 95%	*p*-Value	Adjusted HR	CI of 95%	*p*-Value
RFS	0.913	0.491–1.701	0.775	0.803	0.417–1.547	0.512
Age (per year)	1.068	1.025–1.112	**0.002**	1.093	1.032–1.158	**0.003**
BMI (per kg/m^2^)	1.041	0.995–1.088	0.081	1.072	1.022–1.125	**0.004**
SAPS II at ECMO start	1.036	1.004–1.069	**0.026**	0.999	0.960–1.040	0.971

Bold values indicate a statistical significance (*p* < 0.05). A sensitivity analysis accounting for the competing risk of alive ICU discharge (Fine-Gray) was conducted for RFS (univariable analysis: subdistribution HR 1.05 [0.57–1.92], *p* = 0.88) and (multivariable analysis: HR: 0.91 [0.49–1.71], *p* = 0.77, adjusted for age and BMI). Abbreviations: RFS, refeeding syndrome; HR, hazard ratio; CI, confidence interval; BMI, body mass index; SAPS II, Simplified Acute Physiology Score II; ICU, intensive care unit.

## Data Availability

The data that support the findings of this study are available from the corresponding authors upon reasonable request.

## Supplementary Materials

The following supporting information can be downloaded at: https://www.mdpi.com/article/10.3390/nu18213491/s1, Table S1: Robustness of the main findings across RFS definitions and observation windows.
