# Supplementary material for: Refeeding Hypophosphatemia During ECMO Support: A Retrospective Cohort Study on Incidence, Predictors, and Association with Outcome
Source: Nutrients. 2026 Oct 23;18(21):3491. doi: 10.3390/nu18213491 (PMC13765521; doi:10.3390/nu18213491)
Supplement: Supplementary file 1 [file nutrients-18-03491-s001.zip › nutrients-4621702-supplementary.pdf]

Supplementary Material:

Refeeding Hypophosphatemia During ECMO Support: A Retrospective Cohort Study on Incidence, Predictors, and Association with Outcome

Patrick Haselwanter [1], Max Oberweger [1], Sarah Schwaiger [1], Marlene Hintersteininger [1], Marlene Riedl-Wewalka [1], Mathias Maleczek [2,3], Daniel Laxar [2,3], Martina Hermann [2,3], Nina Buchtele [4], Thomas Staudinger [4], Christian Zauner [1] and Mathias Schneeweiss-Gleixner [1,*]

[1] Department of Medicine III, Division of Gastroenterology and Hepatology, Intensive Care Unit 13H1, Medical University of Vienna, Waehringer Guertel 18–20, 1090 Vienna, Austria
[2] Department of Anesthesia, Intensive Care Medicine and Pain Medicine, Medical University of Vienna, 1090 Vienna, Austria
[3] Ludwig Boltzmann Institute for Digital Health and Patient Safety, Medical University of Vienna, 1090 Vienna, Austria
[4] Department of Medicine I, Intensive Care Unit 13i2, Medical University of Vienna, 1090 Vienna, Austria

Corresponding Author*

Mathias Schneeweiss-Gleixner, M.D., PhD

Department of Medicine III, Division of Gastroenterology and Hepatology, Intensive Care Unit 13H1, Medical University of Vienna

Waehringer Guertel 18-20, 1090 Vienna, Austria

Phone: +43-1-40400-47660. Fax: +43-1-40400-47970

Email: mathias.schneeweiss@meduniwien.ac.at

Supplementary Table

Supplementary Table S1. Robustness of the main findings across RFS definitions and observation windows.

RFS definition	Incidence	ICU mortality			Energy d1-3
	n, (%)	adjusted HR	95% CI	p-value	p-value
Extended Marik (primary, 7-day)[1]	46 (45.5)	0.80	0.42-1.54	0.51	0.35
Extended Marik (14-day window)	54 (53.5)	1.18	0.59-2.36	0.64	0.37
Extended Marik (whole ECMO course)	62 (61.4)	1.13	0.56-2.30	0.73	0.51
Hypophosphatemia-based (<0.65, no fall criterion)	49 (48.5)	0.68	0.36-1.30	0.24	0.10
De novo (fall from normal baseline)*	29 (28.7)	1.01	0.51-2.00	0.97	0.26
Strict Marik, 1996 (fall only)[2]	37 (36.6)	1.12	0.58-2.14	0.74	0.73
ASPEN, 2020 (all electrolytes)[3]	85 (84.2)	1.39	0.54-3.61	0.49	0.46
Doig, 2015[4]	24 (23.8)	0.76	0.35-1.64	0.48	**0.04**

The extended Marik definition (7-day window) served as the primary reference. Incidence under each definition (95% Wilson CI in the text). ICU mortality: Cox model adjusted for age and BMI (event = ICU death, time origin ECMO start). “Energy d1-3, p”: p-value for the comparison of early energy delivery (mean percentage of requirements over MNT days 1-3) between patients with and without RFS under each definition (Mann-Whitney U test).
*The de novo definition requires a qualifying phosphate fall (>0.16 mmol/L to <0.65 mmol/L) arising from a normal baseline (≥0.65 mmol/L on the first day of MNT), thereby excluding all patients with pre-existing hypophosphatemia.

Abbreviations: RFS, refeeding syndrome; aHR, adjusted hazard ratio; CI, confidence interval; MNT, medical nutrition therapy; BMI, body mass index.

References:
[1] *Schneeweiss-Gleixner M, Haselwanter P, Schneeweiss B, Zauner C, Riedl-Wewalka M. Hypophosphatemia after Start of Medical Nutrition Therapy Indicates Early Refeeding Syndrome and Increased Electrolyte Requirements in Critically Ill Patients but Has No Impact on Short-Term Survival. Nutrients. 2024 Mar 22;16(7):922.*

[2] *Marik PE, Bedigian MK. Refeeding hypophosphatemia in critically ill patients in an intensive care unit. A prospective study. Arch Surg. 1996;131(10):1043-7.*

[3] *da Silva JSV, Seres DS, Sabino K, Adams SC, Berdahl GJ, Citty SW, et al. ASPEN Consensus Recommendations for Refeeding Syndrome. Nutr Clin Pract. 2020;35(2):178-95.*

[4] *Doig GS, Simpson F, Heighes PT, Bellomo R, Chesher D, Caterson ID, et al. Restricted versus continued standard caloric intake during the management of refeeding syndrome in critically ill adults: a randomised, parallel-group, multicentre, single-blind controlled trial. Lancet Respir Med. 2015;3(12):943-52.*
